# Effects of Tongmai oral liquid in femoral ateriovenous fistula

**DOI:** 10.1186/s12906-015-0844-8

**Published:** 2015-09-07

**Authors:** Pei-Ling Su, Kun Bao, Han-Guo Peng, Wei Mao, Guan-Su Wang, Ni-Zhi Yang, Wen-Jia Geng, Yi-Qun Lin, Xi-Na Jie

**Affiliations:** Department of Nephrology, The Second Affiliated Hospital, Guangzhou University of Chinese Medicine (Guangdong Provincial Hospital of Chinese Medicine), No.111 of Dade Road, Guangzhou, 510120 China; Department of Nephrology, The Third Affiliated Hospital, Guangxi University of Chinese Medicine (Liuzhou traditional Chinese Medical Hospital), No.32 of Jiefang Road, Liuzhou, 545001 China; Jiangmen Xinhui District Hospital of Chinese Medicine, No.47 of Huimin Road, Jiangmen, 529100 China

**Keywords:** Tongmai oral liquid, Early fistula maturation, Hemodialysis

## Abstract

**Background:**

This study was conducted to investigate the protective effect of Tongmai oral liquid on arteriovenous fistula function and to provide an effective method to promote fistula maturation.

**Methods:**

Fifteen female and fifteen male SPF New Zealand rabbits were randomly allocated into 3 groups including control, Aspirin and Tongmai oral liquid groups. A side-to-side femoral arteriovenous fistula was established in each rabbit and then animals were treated with Aspirin or Tongmai oral liquid for 2 weeks. The concentrations of circulating ET-1 and NO were determined before and after operation (on preoperative day, operative day, post-D1, post-D3, post-D7 and post-D15), respectively. Blood flow of the fistula stoma and contralateral artery and vein was determined on the 15^th^ postoperative day. Last, the fistula stoma was dissected to observe patency, thrombosis and adhesion with surrounding tissues.

**Results:**

28 rabbits survived during the surgical process and the following 15-day observational period. Tissue adhesion of arteriovenous fistula with surrounding tissues was improved and fistula thrombosis was reduced by treatment with Tongmai oral liquid. NO concentration decreased to a different extent after vascular surgery. Tongmai oral liquid failed to regulate the equilibrium between NO and ET-1, but it improved blood flow of fistula stoma, as compared to control and Aspirin groups. Blood flow of fistula stoma in the three groups was lower than that of the contralateral femoral artery.

**Conclusions:**

Tongmai oral liquid improved the function of femoral ateriovenous fistula in the rabbit model by increasing blood flow and reducing thrombosis, probably not by regulating the dynamic equilibrium between NO and ET-1.

## Background

Arteriovenous fistula (AVF) is the first choice for vascular access in hemodialysis patients with chronic kidney diseases. However, it is very difficult to create a functional AVF in senile patients and patients with blood hypertension and diabetes. Moreover, it also costs a lot to maintain AVF and to reduce vascular access-related complications [1]. Because of the frequent failure of maturation of a newly created AVF [2], more and more researchers focus on early fistula maturation.

It has been shown that thrombosis and venous stenosis as a result of neointimal hyperplasia, reduction of blood flow and inadequate dilatation of the venous segment are common causes that contribute to early AVF dysfunction [3–6]. Endothelin-1 (ET-1) and nitric oxide (NO) are mutually antagonistic vasoactive substances secreted by vascular endothelial cell. The dynamic equilibrium of ET-1 and NO is important to maintain normal vascular angiotasis and hemodynamics, and such a balance can regulate endothelial cell proliferation and platelet aggregation [7, 8]. In addition to modulating the contractile state and migration of Vascular Smooth Muscle Cells (VSMC), NO can also inhibits VSMC proliferation [9]. However, the AVF surgery brings injury to endothelial cells and breaks the balance between ET-1 and NO. Besides, chronic kidney disease itself increases neointimal formation and endothelial barrier dysfunction [10]. However, there are no effective methods to improve the early fitula maturation or protect the ateriovenous fistula function so far.

Tongmai oral liquid has been used to treat chronic kidney disease since 1995 in clinical practice of Chinese medicine. It is consisted of radix astragali and radix notoginseng, which are selected based on traditional Chinese medical theory and modern pharmacological studies of Chinese herbal medicine. The main effective ingredients of Tongmai oral liquid are total saponins and total polysaccharides, which can reduce plasma viscosity as well as erythrocyte aggregation index [11]. Our clinical observations showed that it can improve uncomfortable symptoms of patients with glomerulonephritis, such as edema, fatigue, bad appetite, soreness and weakness of waist and knees. And laboratory examinations of the levels of serum creatinine, blood urea nitrogen, 24-h protein excretion, total cholesterol, triglyeride, fibrin degradation product and fibrinogen also decreased to a different extent by treatment with Tongmai oral liquid [12]. Importantly, previous studies showed that Tongmai oral liquid can inhibit platelets’ activation, improve renal hemodynamics, ameliorate hemodialysis patients’ micro-inflammatory state, and improve blood high condensation state [13–16], etc. Therefore, in the present study, we hypothesized that Tongmai oral liquid may potentiate the vascular endothelial cell function and blood flow to promote fistula maturation.

## Methods

### Consent

Approval was obtained from Animal Experimental Ethics Committee of Guangzhou University of Chinese Medicine prior to performing any procedures on animals.

### Experimental animals

The study was carried out in the Laboratory Animal Centre of Guangzhou University of Chinese Medicine. Fifteen female and fifteen male specified-pathogens free (SPF) New Zealand rabbits weighing 2.2-2.4 kg were used (Huadu experimental animal farm, Guangzhou, China, certificate number: 1084322). They were kept in a climate-controlled room with 25 °C and 37 % relative humidity. These rabbits were randomly allocated into 3 groups (control, Aspirin, and Tongmai oral liquid group) according to a random table. Each group consisted of 10 rabbits.

### Drugs

Tongmai oral liquid with bath number of 110303 (consisted of Astragalus Membranaceus 3 g and Notoginseng Radix 1 g per 10 ml) were prepared and provided by the Department of Pharmacology in Guangdong Provincial Hospital of Chinese Medicine, Guangzhou, China. Aspirin (bath number BJ02005, Bayer Healthcare Co. Ltd) was also provided by the Department of Pharmacology in Guangdong Provincial Hospital of Chinese Medicine, Guangzhou, China.

### Administration method and dosage

Administration dosage was determined according to dose convert coefficient table which is applicable to human and animal dose conversion. The convert coefficient W is 2.3 and the formula is D_b_ (mg/kg) = W × D_a_ (mg/kg) [17]. Namely, the administration dose of B animal is equal to W multiplied by that of A animal. Human’s dosage of Tongmai oral liquid is 60 ml per day, and Aspirin dosage is 100 mg per day.

According to this dosage conversion, solution of Aspirin was made with concentration of 0.77 mg/ml and was administered to rabbits in Aspirin group by 5 ml/kg. The control group was given normal saline (5 ml/kg). The experimental group was given Tongmai oral liquid (5 ml/kg). These drugs were all given by intragastric administration on the day after AVF operation for 14 days.

### Establishment of femoral arteriovenous fistula

After randomly allocated, rabbits were anesthetized with pentobarbital sodium (3 % volume fraction, 1 ml/kg) via ear vein injection. Subsequently, they were fixed in a supine position with left leg extended, shaved and sterilely prepared. Through a skin incision 2 cm under the left groin ligament, femoral artery and femoral vein were dissected and exposed. All branches of the femoral artery and vein were ligated. The two vessels were both clamped proximally and distally. A 5–6 mm long incision was made on adjoining sides of the artery and vein respectively and heparin saline (100 IU/ml) was used to syringe the wounds. Then the wounds were saturated with 12–0 atraumatic suture to form a side-to-side femoral artery to vein anastomosis. Next, the vascular clamps were loosed and anastomotic vessels were patent. Finally, the skin was closed with interrupted suture using 4–0 suture and covered with erythromycin ophthalmic ointment to prevent infection.

### Determinations of circulating ET-1 and NO concentration

Blood samples (2 ml) were collected from central auricular artery in each group on preoperative day, operative day, post-D1, post-D3, post-D7 and post-D15. ET-1 concentration was determined by using 96 T rabbit ET-1 enzyme linked immunosorbent assay (ELISA) kit (American R&D systems, Batch No.CSB-E06951Rb) [18]. NO concentration was determined by using 96 T rabbit NO ELISA kit (American R&D systems, Batch No.KGE001) [18].

### Measurement of blood flow

Blood flow of fistula stoma was determined by using an ultrasonic flow probe (Transonic System, Ithaca, NY) on the 15^th^ postoperative day, following exposure of anastomotic vessels. Blood flow was recorded every 5 s, and the totally observational time was 1 min. Meanwhile, this procedure was also used to test blood flow of the contralateral femoral artery and vein at the same position. The fistula stoma was next dissected to observe patency, thrombosis and adhesion with surrounding tissues. Finally, the animals were executed by euthanasia.

### Statistical analysis

Data are reported as mean ± SD. Data were analyzed by one-way ANOVA or repeated measures analysis of variance. P-values of <0.05 were regarded as significant. All calculations were made with SPSS 17.0 software.

## Results

### Surgical procedure

Two rabbits in the control group died during the surgical procedure. One died of overdose of anesthesia. The other was executed because it regained consciousness during the operation process and was struggled strongly to break the operative vessels. Finally, AVFs were successfully established in 28 rabbits, with 8 in control group, 10 in Aspirin group, and another 10 in Tongmai group. All the 28 rabbits survived during the15-day observational period.

### Fistula observations

As shown in Table 1, in Tongmai group, there was one small thrombosis in one fistula stoma with decreased blood flow, while in the other two groups two stomas were completely blocked by thrombosis, with no blood flow. Compared with Aspirin and control groups, tissue adhesion in Tongmai group was significantly reduced by 30 and 60 % respectively. These observations indicated that Tongmai oral liquid could prevent thrombosis and reduce tissue adhesion of AVF with surrounding tissues.

**Table 1:** Fistula observations in each group on the 15^th^ postoperative day

	Number	Fistula observations	
Groups	(n)	Tissue adhesion	Thrombosis
Control group	8	8	2
Aspirin group	10	7	2
Tongmai group	10	4	1

### NO and ET-1 concentration measurements

**Fig. 1:**
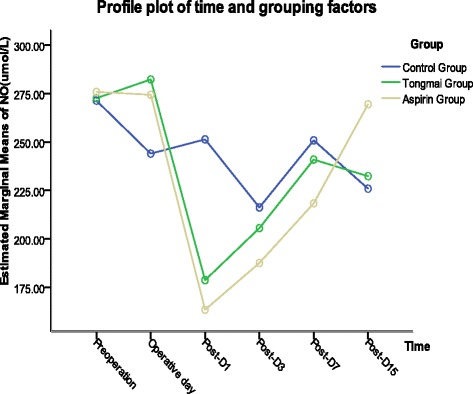
Change of NO concentrations during the observational period

The concentrations of NO and ET-1 were determined before and after operation, as shown in Tables 2 and 3. NO concentrations were significantly different at the indicated time points after surgery (F = 19.267, *P* = 0.000), both in Aspirin group (F = 1.167, *P* = 0.005) and Tongmai group (F = 59.258, *P* = 0.002), except that in control group (F = 1.207, *P* = 0.326). During the observational period, NO concentration was decreased to a different extent in each group following vascular surgery, which was in accordance with previous literatures [19]. The change of NO concentration was of no significance among three groups (F = 0.817, *P* = 0.453). Interaction effect existed between different treatment methods and time points (F = 4.299, *P* = 0.002). After a transient decline (lowest on post-D1), NO concentration increased gradually during the following observational days, as illustrated in Fig. 1.

**Table 2:** Changes of NO concentration (umol/L, $\overline{x}$ ± S)

Group	N		Before or After operation						*F*	*P*
			Preoperat-ive day	Operative day	Post-D1	Post-D3	Post-D7	Post-D15		
Control group	8	$\overline{x}$	271.31	243.97	251.34	216.21	250.84	225.90	1.207	0.326
		*s*	18.17	14.49	56.81	55.82	63.18	79.20		
Aspirin group	10	$\overline{x}$	275.85	274.34	163.44	187.58	218.32	269.46	1.167	0.005
		*s*	14.39	10.78	20.65	27.74	14.23	24.45		
Tongmai group	10	$\overline{x}$	272.57	282.24	178.70	205.55	240.90	232.32	59.258	0.000
		*s*	22.12	17.95	32.11	23.35	28.67	84.64		
Sum	28	$\overline{x}$	273.38	268.49	194.00	202.18	235.67	243.75	19.267*	0.000*
		*s*	17.93	21.79	52.24	37.25	39.58	67.83		
*F*	0.817*		0.148	16.269	13.297	1.419	1.724	0.150	(*F = 4.299 P = 0.002*) **	
*P*	0.453*		0.863	0.000	0.000	0.261	0.199	0.333		

* F statistic and P value of main effect; ^b^ F statistic and P value of interaction effect

**Table 3:** Changes of ET-1 concentration (pg/ml, $\overline{x}$ ± S)

Group	n		Before or After operation						*F*	*P*
			Preopera-tive day	Operative day	Post-D1	Post-D3	Post-D7	Post-D15		
Control group	8	$\overline{x}$	7.66	7.50	7.57	7.75	8.42	7.78	4.298	0.004
		*s*	0.54	0.47	0.53	0.60	0.58	0.59		
Aspirin group	10	$\overline{x}$	7.59	7.30	7.44	7.55	7.44	7.37	1.690	0.156
		*s*	0.57	0.34	0.35	0.54	0.44	0.49		
Tongmai group	10	$\overline{x}$	7.50	7.63	8.01	7.81	7.76	7.59	0.659	0.656
		*s*	0.50	0.45	0.56	0.34	0.59	0.55		
Sum	28	$\overline{x}$	7.58	7.47	7.68	7.70	7.84	7.57	2.669*	0.025*
		*s*	0.52	0.43	0.53	0.49	0.65	0.55		
*F*	3.179*		0.217	1.541	3.796	0.746	7.500	1.342	*(F* = 2.274 *P* = 0.018)**	
*P*	0.059*		0.806	0.234	0.036	0.485	0.003	0.280		

* F statistic and P value of main effect; ** F statistic and P value of interaction effect

**Fig. 2:**
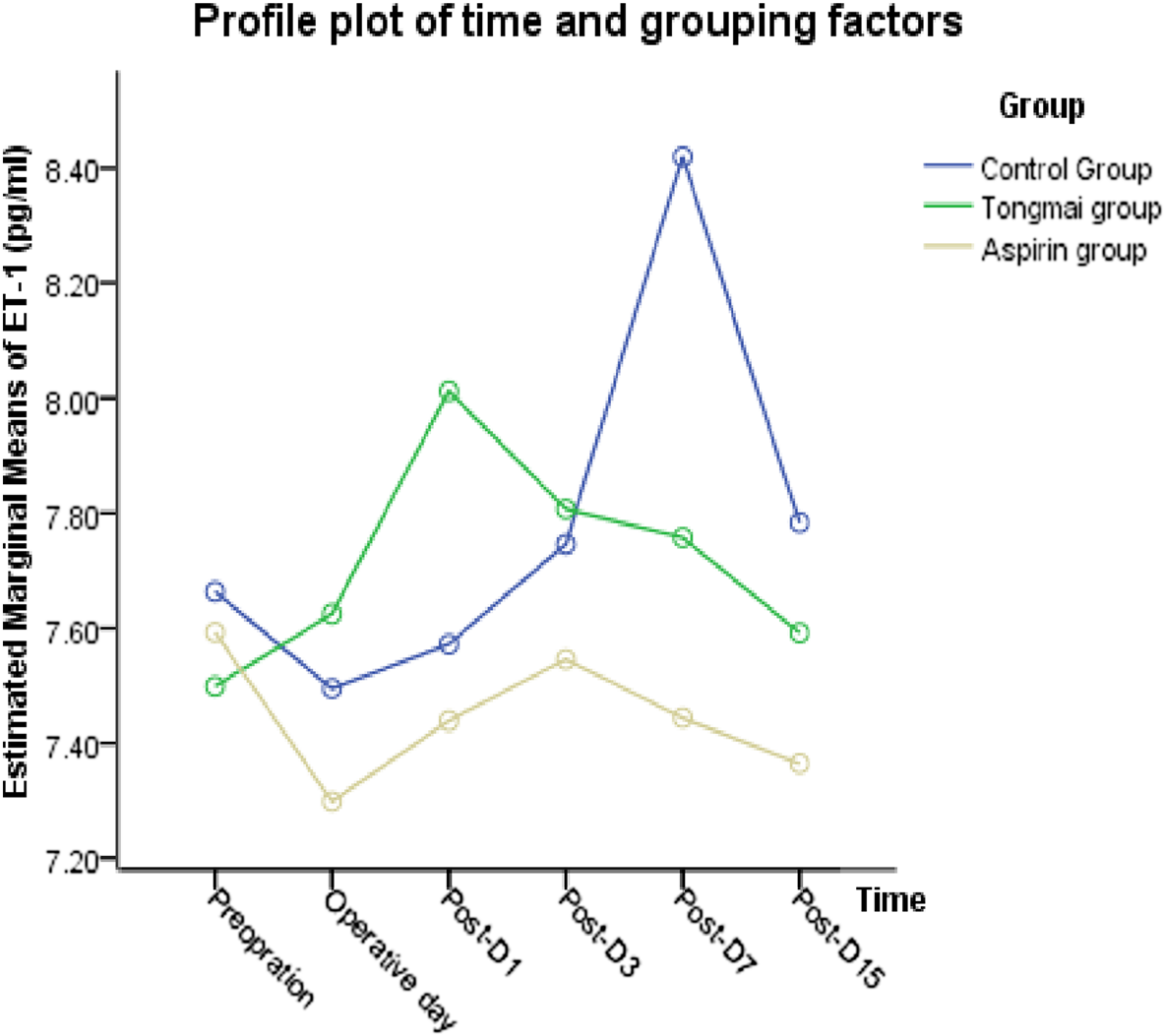
Change of ET-1 concentrations during the observational period

ET-1 levels were significantly different after surgery (F = 2.669, *P* = 0.025), especially in control group (F = 4.298, *P* = 0.004), while of no significance in both Aspirin group (F = 1.690, *P* = 0.156) and Tongmai group (F = 0.659, *P* = 0.656). The change of ET-1 concentration was not significant among the three groups (F = 3.179, *P* = 0.059). Interaction effect existed between different treatment methods and time points (F = 2.274, *P* = 0.018). After surgery, ET-1 levels in Tongmai and control groups increased to a peak on post-D1 and post-D7 respectively, and subsequently decreased in both groups, as demonstrated in Fig. 2.

### Blood flow measurements

Blood flow of fistula stoma, contralateral femoral artery and vein was measured on the 15^th^ postoperative day. Fistula blood flow was significantly lower than that of the contralateral femoral artery in the same group (*P* < 0.05). And this outcome was mildly improved by Tongmai oral liquid although there was no significance among three groups (F = 1.668, *P* = 0.209). On the other hand, blood flow of the contralateral femoral artery was at similar level among three groups (*P* > 0.05), indicating that Aspirin and Tongmai oral liquid did not play a role in the healthy vessel (Table 4).

**Table 4:** Blood flow measurements on the 15^th^ postoperative day

	Number	Blood flow (ml/min)		
Groups	(n)	Contralateral femoral artery	Contralateral femoral vein	Fistula stoma
Control group	8	10.63 ± 1.99	8.25 ± 1.04	6.00 ± 3.89*^,^ **
Aspirin group	10	11.12 ± 1.91	8.40 ± 2.27	6.00 ± 3.49*^,^ **
Tongmai group	10	11.30 ± 1.71	8.91 ± 1.79	8.80 ± 4.24*^,^ **

*P < 0.05 compared with blood flow of contralateral artery in the same group

**Blood flow of the three groups were analyzed by one-way analysis of variance (F = 1.668, *P* = 0.209)

## Discussion

Thrombosis and venous stenosis as a result of neointimal hyperplasia are leading causes for AVFs dysfunction [3, 4]. Mechanisms of neointimal hyperplasia are related to endothelial cell damage, inflammation, decrease of NO synthesis, migration of myofibroblasts, oxidative stress and laminar fluid shear stress [20]. In addition, upregulation of transforming growth factor beta1 (TGF-beta1) plays an important role in the process of neointimal hyperplasia by promoting fibroblasts proliferation [21, 22] and stimulating adventitial fibroblasts transforming to myofibroblasts and migration [23]. Besides, surgical trauma to the vasa vasorum of the outflow vein can promote adventitial myofibroblasts migration as well as lead to neointimal hyperplasia [24]. Of note, our previous studies showed that Tongmai oral liquid can suppress production of TGF-beta1 in diabetic nephropathy murine models and mesangial proliferative glomerulonephritis rats [25, 26]. Therefore, we hypothesized that Tongmai oral liquid is applied for improving AVFs dysfunction.

In traditional Chinese medical theory, the complex process of neointimal hyperplasia and stenosis is, at least in part, due to Qi-deficiency and the subsequent blood stasis. Qi-deficiency and blood stasis exists in the progression of chronic kidney disease [27], and the surgical procedure of establishing an arteriovenous fistula aggravates injuries of blood vessels and Qi activities. Modern studies of Chinese medicine consider blood stasis as blood circulation obstacle, such as thrombosis and platelet aggregation [28], which is closely related to endothelial cell injury, high blood viscosity and inflammatory response [29–31]. Tongmai oral liquid was made from several Chinese herbals (Table 5) which can relieve blood stasis via nourishing Qi. For example, the pharmacological studies have shown that both astragali and radix notoginseng can improve hemorrheology, anti-platelet aggregation, and promote fibrinolysis to reduce thrombosis [32–35]. Moreover, we previously reported that Tongmai oral liquid is able to ameliorate hemodialysis patients’ micro-inflammatory state and inhibit platelets’ activation [13, 15]. Furthermore, as shown in Table 4, Tongmai oral liquid could increase blood flow of fistula stoma, compared with Aspirin and control group (8.80 ± 4.24 vs 6.00 ± 3.49 vs 6.00 ± 3.89 ml/min). In rabbit AVF model, 2 weeks’ treatment of Tongmai oral liquid could improve tissue adhesion around the AVF and reduce thrombosis. As shown in Table 1, tissue adhesion in Tongmai group was significantly reduced by 30 and 60 % respectively compared with Aspirin and control groups. Aspirin treatment served as positive control, since it is an effective antiplatelet aggregation drug to prevent fistula thrombosis [36] and improve AVF survival [37]. Aspirin’s effect of anti-thrombosis seemed not to be effective enough in this model, which could be due to reduction of blood flow. Altogether, our results strongly suggest that Tongmai oral liquid can promote a well functional AVF through, at least in part, increasing blood flow as well as suppressing neointimal hyperplasia.

**Table 5:** Components of Tongmai Oral Liquid (per 10 ml)

Latin binomial name	English name	Part used	Type of product	Weight (g)
Astragalus Membranaceus	Membranous Milkvetch Root	Root	Raw	3
Notoginseng Radix	Sanchi	Root	Raw	1

It is well known that ET-1 is a powerful vasoconstrictor while NO is a potent vasodilative substance [7]. The impairment of the balance between NO and ET-1 can induce endothelial cell dysfunction [8]. To determine if Tongmai oral liquid could promote blood circulation by improving endothelial cell function, concentrations of NO and ET-1 were evaluated in our model studied. Unfortunately, the data showed that Tongmai oral liquid and Aspirin only slightly reversed the downward trend of NO after operation, which was of no statistical significance.

Interestingly, in our rabbit AVF models, blood flow of fistula stoma was generally low in each group, even lower than that of the contralateral femoral artery which is contrary to clinical practice and other animal AVF models [38–43]. Though reduction of fistula blood flow is frequently in the period of fistula maturation and maintains, both clinical and experimental studies demonstrated increased blood flow of fistula and cardiac output as well as decreased systematic vascular resistance following the creation of AVF. Since it is a newly created AVF with successful surgical procedure, the following aspects are possibly to be considered for contributing to low blood flow: 1) Surgery itself is an injurious strike to normal blood vessel anatomy to cause pathological changes of blood milieu internae and hemodynamics. 2) Stenosis of fistula stoma and out-flow vein tract caused by neointimal hyperplasia and vascular remodeling. A previous study in a rodent femoral artery to vein fistula [44] demonstrated apparently polypoid neointimal hyperplasia at the anastomosis venous lesion on the 14^th^ day after surgery. 3) Underlying thrombosis. 4) The observational period was too short for a fistula to maturate. Therefore, it is worth doing the further studies in our model established to clarify all the questions raised, especially to detect the role of Tongmai oral liquid in venous stenosis.

There are also some limitations in our study. Since Tongmai oral liquid has special colour and taste which can be distinguished from other drugs easily by the experimenter, double blind method is difficult to implement during the experimental process. Besides, though sample size of the study was determined based on experimental estimation, it still seems not sufficient enough. We plan to expand sample size and try to overcome the technical problem of implementing blinding method in our further study.

## Conclusions

In conclusion, Tongmai oral liquid improved the function of femoral AVF in the rabbit model by increasing blood flow and reducing thrombosis. The mechanisms of this protective effect are probably not relevant to regulating the dynamic equilibrium between NO and ET-1. Further studies are needed to examine the role of Tongmai oral Liquid in venous stenosis in rabbit AVF model.

## Abbreviations

AVF: Ateriovenous fistula
ELISA: Enzyme linked immunosorbent assay
ET-1: Endothelin-1
NO: Nitric oxide
SPF: Specified-pathogens free
TGF-beta1: Transforming growth factor beta1
VSMC: Vascular smooth muscle cells
